# Safety and immunogenicity of Innovax bivalent human papillomavirus vaccine in girls 9–14 years of age: Interim analysis from a phase 3 clinical trial

**DOI:** 10.1016/j.vaccine.2024.02.077

**Published:** 2024-04-02

**Authors:** Khalequ Zaman, Anne E Schuind, Samuel Adjei, Kalpana Antony, John J Aponte, Patrick BY Buabeng, Firdausi Qadri, Troy J Kemp, Lokman Hossain, Ligia A Pinto, Kristen Sukraw, Niranjan Bhat, Tsiri Agbenyega

**Affiliations:** a Infectious Diseases Division, International Centre for Diarrhoeal Disease Research, Dhaka, Bangladesh; b PATH, Center for Vaccine Innovation and Access, Seattle, Washington, United States; c Malaria Research Center, Agogo Presbyterian Hospital/Kwame Nkrumah University of Science and Technology, Agogo, Ghana; d HPV Serology Laboratory, Vaccine, Immunity, and Cancer Directorate, Frederick National Laboratory for Cancer Research, Frederick, Maryland, United States

**Keywords:** Human papillomavirus (HPV), HPV vaccine, Immunization, Immunobridging, One-dose immunogenicity

## Abstract

**Background:**

World Health Organization human papillomavirus (HPV) vaccination recommendations include a single- or two-dose schedule in individuals 9–20 years old and advice for generating data on single-dose efficacy or immunobridging. The ongoing Phase 3 trial of Innovax’s bivalent (types 16 and 18) HPV vaccine (Cecolin®) assesses in low- and middle-income countries alternative dosing schedules and generates data following one dose in girls 9–14 years old. Interim data for the 6-month dosing groups are presented.

**Methods:**

In Bangladesh and Ghana, 1,025 girls were randomized to receive either two doses of Cecolin at 6-, 12-, or 24-month intervals; one dose of Gardasil® followed by one dose of Cecolin at month 24; or two doses of Gardasil 6 months apart (referent). Serology was measured by enzyme-linked immunosorbent assay (ELISA) and, in a subset, by neutralization assays. Primary objectives include immunological non-inferiority of the Cecolin schedules to referent one month after the second dose. Safety endpoints include reactogenicity and unsolicited adverse events for 7 and 30 days post-vaccination, respectively, as well as serious adverse events throughout the study.

**Results:**

Interim analyses included data from the two groups on a 0, 6-month schedule with 205 participants per group. One month after Dose 2, 100% of participants were seropositive by ELISA and had seroconverted for both antigens. Non-inferiority of Cecolin to Gardasil was demonstrated. Six months following one dose, over 96% of participants were seropositive by ELISA for both HPV antigens, with a trend for higher geometric mean concentration following Cecolin administration. Reactogenicity and safety were comparable between both vaccines.

**Conclusions:**

Cecolin in a 0, 6-month schedule elicits robust immunogenicity. Non-inferiority to Gardasil was demonstrated one month after a 0, 6-month schedule. Immunogenicity following one dose was comparable to Gardasil up to six months. Both vaccines were safe and well tolerated (ClinicalTrials.gov No. 04508309).

## Highlights

• Innovax bivalent HPV vaccine (Cecolin), and Gardasil have similar safety profiles.
• Two Cecolin doses six months apart are immunologically non-inferior to Gardasil.
• Six months after one dose, Cecolin is highly immunogenic.
• Cecolin expands the options for HPV vaccination in low- and middle-income countries.

## 1 Introduction

Human papillomavirus (HPV) is a very common sexually transmitted infection, with more than 90 % of sexually active men and 80 % of sexually active women being infected with HPV in their lifetime [1]. In 2020, cervical cancer was the fourth most common cancer among women globally, with 604,127 new cases and 341,831 deaths, but was the second most common female cancer in young women 15–44 years of age [2]. Over 90 % of deaths due to invasive cervical cancer occur in low- and middle-income countries (LMICs) and, thus, cervical cancer remains a disease of geographic and economic inequity [3]. Persistent infection with oncogenic HPV types is well established as a prerequisite cause of cervical cancer and other anogenital cancers, as well as head and neck cancers. HPV-16 and HPV-18 together are responsible globally for 71 % of cervical cancer cases and approximately 90 % of cancers at other anatomical sites, such as the anus and oropharynx [4].

Currently, four highly effective and safe HPV vaccines have obtained World Health Organization (WHO) prequalification: Gardasil® and Gardasil®9 (Merck & Co), Cervarix® (GlaxoSmithKline), and Cecolin® (Xiamen Innovax Biotech Co. Ltd.). Cecolin contains 40 μg of HPV-16 and 20 μg of HPV-18 recombinant L1 virus-like particles (VLPs) expressed in *Escherichia coli* and absorbed to aluminum hydroxide adjuvant [5]. Cecolin is recommended as a two- or three-dose regimen for girls 9–14 years of age and a 3-dose regimen above 14 years of age.

Despite HPV vaccines being available since 2006, only 15 % of girls below 15 years of age are fully vaccinated [6]. The uptake has been slower than expected in LMICs due to vaccine and program costs, global supply constraints, and logistical barriers to delivering a two-dose vaccination schedule to young girls. In August 2020, the World Health Assembly adopted a global strategy to accelerate the elimination of cervical cancer as a global health problem through a three-pronged approach: vaccination, screening, and treatment. With respect to vaccination, the goal is to achieve HPV vaccination in 90 % of girls by 15 years of age by 2030 [7].

In December 2022, WHO updated its position paper for HPV vaccines to recommend a 2-dose schedule with a dosing interval of 6 to 12 months for the primary target group from 9 years of age. For all older age groups for which HPV vaccines are licensed, WHO recommended considering longer intervals if programmatically useful [8]. Furthermore, an alternative single-dose schedule from 9 to 20 years of age is also included in the WHO recommendations based on evidence suggesting that a single dose has comparable efficacy and duration of protection to a multi-dose schedule. For single-dose schedules, WHO advises the need for demonstration of efficacy or successful immunobridging (i.e., evidence that antibody levels at peak and plateau phases are comparable to those of vaccines with proven single-dose efficacy) [8].

The current randomized, controlled trial was initiated to evaluate Cecolin—a bivalent HPV vaccine recently WHO prequalified—in alternate 2-dose regimens, compared with a widely used HPV vaccine, Gardasil, in African and South Asian girls aged 9–14 years. The study generates data in a broader geographic representation than prelicensure trial data, particularly in low-resource populations, as well as data on 2-dose extended schedules and vaccine interchangeability. Furthermore, the study will also generate data following single-dose Cecolin administration up to 24 months post–vaccine administration. This report presents the data from an interim analysis assessing Cecolin’s safety and immunological non-inferiority to that of Gardasil, both administered on a 0, 6-month schedule. Data related to other study vaccination schedules, as well as persistence of the immune response following a 0, 6-month schedule, will be evaluated at study end and presented in a subsequent manuscript.

## 2 Material and methods

### 2.1 Study design

The study was an open-label, randomized, Gardasil-controlled Phase 3 clinical trial assessing the non-inferiority of bivalent Cecolin vaccine in healthy girls 9–14 years of age. The trial was registered before the onset of patient enrollment under ClinicalTrials.gov NCT No. 04508309. The study was conducted in one clinical site in Bangladesh and one clinical site in Ghana. Safety and immunogenicity of Cecolin on different dosing regimens were assessed using Gardasil as a comparator.

The study was conducted in accordance with the ethical principles set forth in the World Medical Association Declaration of Helsinki and Good Clinical Practice guidelines. The study was approved by WIRB-Copernicus Group Institutional Review Board, as well as by ethics committees in both countries. Regulatory approvals were also obtained from the regulatory authorities in Bangladesh and Ghana.

### 2.2 Study population

A total of 1,025 girls aged 9–14 years participated in the study, of which 675 and 350 were recruited at the study sites in Bangladesh and Ghana, respectively. Written informed consent and assent (if applicable) were obtained from parents or legally acceptable representatives, and participants, respectively. Eligible participants were healthy girls by clinician medical history and physical examination, aged 9–14 years and living in reasonable proximity to the study site, without plans to leave the area prior to the end of the study, and able and willing to provide assent in addition to parental consent. The key exclusion criteria were previous HPV vaccination, pregnancy, immunocompromising conditions (including HIV infection), and presence of an acute disease on the day of vaccination.

### 2.3 Study vaccines

Two WHO-prequalified prophylactic HPV VLP vaccines containing L1 major capsid proteins and aluminum-adjuvanted were evaluated in the study. Cecolin is a bivalent VLP-based vaccine composed of L1 proteins produced in a recombinant *E. coli* expression system and adjuvanted with aluminum hydroxide. Each dose of the vaccine contains 40 µg HPV-16 and 20 µg HPV-18 L1 proteins. The comparator vaccine, Gardasil, is a recombinant quadrivalent vaccine produced in *Saccharomyces cerevisiae* and adjuvanted with amorphous aluminum hydroxyphosphate sulfate. Each dose of the vaccine contains approximately 20 µg of HPV-6, 40 µg of HPV-11, 40 µg of HPV-16, and 20 µg of HPV-18 L1 proteins.

Study vaccines were administered by intramuscular route with the preferred site for vaccination being the deltoid muscle of the upper arm.

### 2.4 Clinical procedures

After informed consent and assent were obtained, study participants were screened for eligibility, including a medical history with clinical examination and a urine pregnancy test in girls of child-bearing potential (i.e., having attained menarche). Eligible participants were invited for enrollment either on the same day or within 28 days of the screening visit and were randomly allocated to one of the five study arms.

Participants were block randomized 1:1:1:1:1 using the Interactive Web Response System component of the database system and stratified by site to one of the five study arms composed of a 2-dose regimen of Cecolin on a 0, 6-; 0, 12-; or 0, 24-month schedule, or a 2-dose regimen of Gardasil on a 0, 6-month schedule, or a first dose of Gardasil followed 24 months later by a single dose of Cecolin.

Blood samples were collected for serology at baseline, prior to Dose 2 administration and one month post–Dose 2, as well as for the two study groups on a 0, 6-month schedule at study month 24. Solicited injection-site (pain, swelling, erythema, induration, pruritus, and abscess) and systemic (rash, axillary temperature, headache, vomiting, nausea, fatigue, chills, muscle pain, cough, diarrhea, dizziness, allergic dermatitis, syncope, and anorexia) adverse events (AEs) were recorded after each vaccination daily for the next 7 days through home visits. Parents were also asked to contact the study team with any health-related concerns, participants were provided necessary treatment, and the study team recorded all unsolicited AEs until 30 days post-vaccination. The severity of AEs was assessed based on the *Division of AIDS Table for Grading the Severity of Adult and Pediatric Adverse Events*, Corrected Version 2.1, July 2017, US National Institutes of Health. The severity grading criteria grade AEs from mild (grade 1) to life-threatening (grade 4). Grade 5 was assigned to any AE leading to death. Windows for study visits were predefined in the protocol.

A protocol safety review team provided safety oversight and consisted of the site principal investigators, a sponsor medical officer, and a clinical research organization medical monitor to routinely monitor safety throughout the duration of the trial. In addition, a Data and Safety Monitoring Board, formed by independent vaccine, pediatric, and infectious disease experts, as well as a biostatistician, was established to periodically review cumulative data and ensure safeguarding of the interests of trial participants by assessing safety and monitoring the overall conduct of the clinical trial.

### 2.5 Immunological assays

Samples were tested at the Frederick National Laboratory for Cancer Research in Frederick, Maryland, United States, by L1 VLP ELISA for anti-HPV-16 and anti-HPV-18 immunoglobulin G (IgG) antibodies in all participants and by pseudovirion-based neutralization assay (PBNA) for anti-HPV-16 and anti-HPV-18 neutralizing antibodies in a subset of participants. HPV-16 and HPV-18 VLPs and pseudovirion particles were produced in a mammalian cell system, independent of the vaccine manufacturer’s production system, as previously described in detail with a few minor changes [9]; additional details are provided in Appendix A. The laboratory did not have access to the randomization list and thus testing was performed in a blinded manner, without knowledge of the treatment allocation. Antibody seropositivity was defined as antibody levels equal to or greater than the assay thresholds (for IgG concentrations: 1.41 IU/mL for HPV-16 and 1.05 IU/mL for HPV-18; for neutralizing antibodies: titers equal to or higher than 21 for HPV-16 and 16 for HPV-18). Seroconversion was defined as a four-fold rise in antibody titers. To evaluate seroconversion, half of the assay threshold was assigned to subjects with values below the assay threshold.

Both assays have been described previously [10] and additional details are provided in Appendix A.

### 2.6 Statistical methods

The three co-primary study objectives were to demonstrate the non-inferiority of Cecolin (administered on 0, 6-month; 0, 12-month; and 0, 24-month 2-dose regimens) to Gardasil (administered on a 0, 6-month 2-dose regimen) based on HPV IgG antibody levels measured one month after the last dose for HPV types 16 and 18. This report presents the primary outcome for Cecolin on a 0, 6-month schedule.

To control the type 1 error for the three co-primary non-inferiority hypotheses, a Bonferroni correction corresponding to 98.3 % confidence intervals (CI) was applied. A sample size of 205 participants was required, accounting for the simultaneous assessment of serotypes HPV-16 and HPV-18 to achieve 95 % power for each comparison and 90 % power overall for the primary objectives assuming a log10 standard deviation of 0.65, 10 % baseline seropositivity, and 15 % of dropout. Non-inferiority was to be demonstrated if the lower bound of the 98.3 % CI of the geometric mean concentration (GMC) ratio was greater than the non-inferiority margin of 0.5 for both HPV-16 and HPV-18. The GMC ratios and corresponding confidence limits were calculated using linear models of the log concentration values adjusted by site and transformed back to the original scale.

The primary immunogenicity analyses were conducted in the per protocol population (i.e., in participants who received two doses of HPV vaccine and had the post-vaccination sample taken in the protocol-defined window), with no major protocol deviations and who were HPV antibody negative at baseline for the type considered in the analysis. The per protocol population includes all eligible participants’ data up to the time of a disqualifying event. The safety analysis was performed in the total vaccinated population and included all participants who received at least one dose of study vaccine.

Analyses of the antibody levels by PBNA, as well as safety assessments, were descriptive in nature. All analyses were performed in SAS® statistical software version 9.4 (SAS Institute Inc., Cary, North Carolina, United States).

## 3 Results

A total of 1,031 girls were screened for eligibility, with 6 being excluded due to not providing consent (n = 1) and failing eligibility criteria (n = 5).

Between March 15 and November 18, 2021, 1,025 girls 9–14 years of age were enrolled in the clinical study and randomly assigned to one of the five treatment groups to receive either two doses of Cecolin 6, 12, or 24 months apart; two doses of Gardasil 6 months apart; or one dose of Gardasil followed 24 months later by one dose of Cecolin. About one-third of the participants (N = 350) were recruited at the Malaria Research Center in Agogo, Ghana, and two-thirds (N = 675) at the International Centre for Diarrhoeal Disease Research (icddr,b) in Matlab, Bangladesh.

**Fig. 1:**
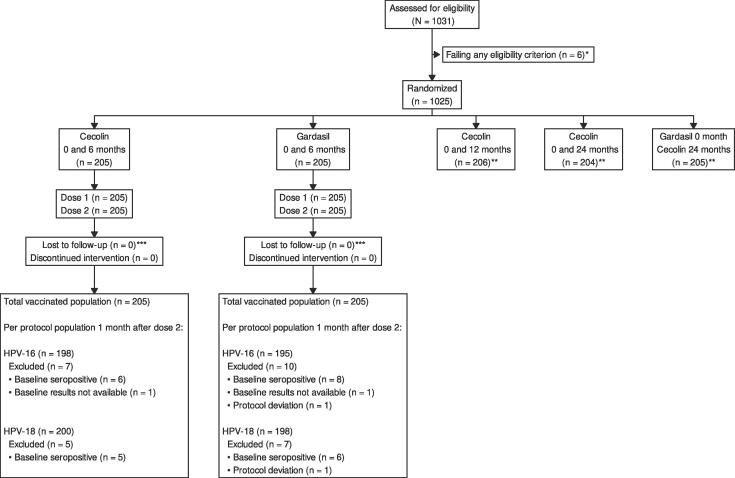
**Consort diagram** * Reasons for screening failure: One subject withdrew consent during the screening process; one subject was pregnant; one subject had history of a physical, mental, or developmental disorder that may hinder ability to comply with the study requirements; two subjects received an investigational product within 30 days prior to randomization or were planning to participate in another research study involving investigational product during the conduct of this study; and one subject had a condition that in the opinion of the investigator would jeopardize the safety or rights of the participant or would render the participant unable to comply with the protocol. ** Groups not included in this interim analysis. *** Lost to follow-up at interim analysis data lock.

For the two groups on a 6-month vaccination schedule included in the interim analysis, compliance with the vaccination schedule was high with all 410 (100 %) of participants in those groups receiving the two-dose vaccination regimen within the protocol-defined window. All participants completed the study month 7 (one month post–Dose 2) follow-up visit and had blood collected for serology assessment (see Fig. 1, Consort diagram).

Baseline characteristics (Table 1) were similar between the two groups with a median age of 11.3 years (interquartile range 10.0; 12.0); 270 (66 %) of girls were Asian and 140 (34 %) were black. Overall, 14 (3.4 %) and 11 (2.7 %) of participants were seropositive for HPV-16 and HPV-18, respectively, at baseline.

**Table 1:** Demographics of participants (total vaccinated cohort).

	Cecolin0, 6-month schedule	Gardasil0, 6-month schedule	All subjects
**Subjects (N)**	205	205	410
**Age (years)**			
Mean (SD)	11.4 (1.46)	11.3 (1.39)	11.3 (1.43)
Median	11	11	11
Min, Max	9, 14	9, 14	9, 14
**Race (%)**			
Asian	66	66	66
Black	34	34	34
**Weight (kg)**			
Mean (SD)	37.2 (10.4)	36.9 (9.5)	37.1 (10.0)
Median	35.5	35.4	35.4
Min, Max	20, 71	20, 77	20, 77
**Height (cm)**			
Mean (SD)	145.5 (10.4)	145.1 (9.3)	145.3 (9.9)
Median	147	146	146
Min, Max	117, 168	120, 165	117, 168
**Country (%)**			
Bangladesh	66	66	66
Ghana	34	34	34

N: number of subjects, SD: standard deviation.

For the interim analysis, 393 (95.9 %) participants were included in the per protocol evaluation for HPV-16 immunogenicity evaluation by ELISA and 398 (97.1 %) for HPV-18 immunogenicity evaluation by ELISA. The PBNA subset included a total of 82 (41 participants per group), of which 80 and 81 contributed to the HPV-16 and HPV-18 evaluation, respectively.

**Fig. 2:**
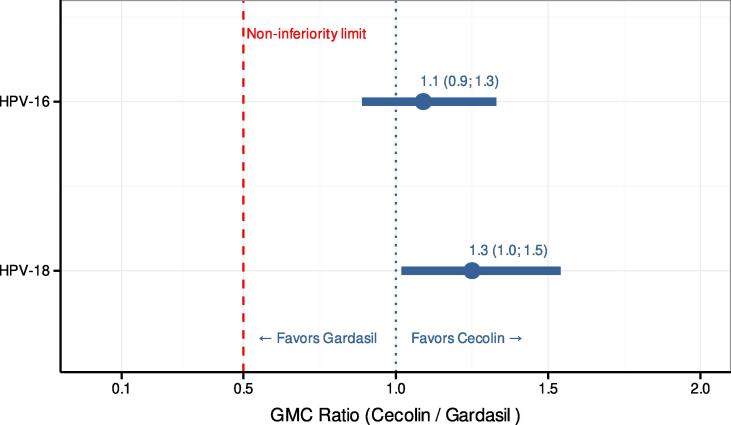
**Non-inferiority evaluation of Cecolin 0–6 months 1 month post–Dose 2.** HPV-16 and HPV-18 geometric mean concentration (GMC) ratio one month post–Dose 2 between Cecolin 0–6 months and Gardasil 0–6 months with 98.3 % confidence intervals. Non-inferiority is demonstrated as both lower limits of the confidence intervals are above the predefined non-inferior limit.

One month post–Dose 2, all participants were seropositive, and the seroconversion rate was 100 % for both HPV antigens. IgG GMC (ELISA) following a two-dose regimen of Cecolin and Gardasil were 1,567 and 1,444 IU/mL, respectively, for HPV-16, and 424 and 336 IU/mL, respectively, for HPV-18 (Table 2). The GMC ratio (Cecolin over Gardasil) was 1.1 (98.3 % CI [0.9; 1.3]) for HPV-16 and 1.3 (98.3 % CI [1.0; 1.5]) for HPV-18. Non-inferiority was demonstrated since the lower limit of the 98.3 % CI was above the pre-specified limit (Fig. 2).

**Table 2:** ELISA HPV-16 and HPV-18 IgG antibody responses (per protocol population).

	Cecolin 0, 6-month schedule			Gardasil 0, 6-month schedule	
**ELISA HPV-16**					
*6 months after Dose 1*					
Subjects (N)	198			196	
Seropositivity rate % (95 % CI)	100	(98.2; 100)		99.5	(97.2; 100)
Seroconversion rate % (95 % CI)	98.5	(95.6; 99.7)		96.9	(95.6; 98.9)
GMC IU/mL (95 % CI)	18.2	(16.3; 20.4)		12.0	(10.8; 13.3)
*1 month after Dose 2*					
Subjects (N)	198			195	
Seropositivity rate % (95 % CI)	100	(98.2; 100)		100	(98.1; 100)
Seroconversion rate % (95 % CI)	100	(98.2; 100)		100	(98.1; 100)
GMC IU/mL (95 % CI)	1567	(1390; 1767)		1444	(1287; 1621)
**ELISA HPV-18**					
*6 months after Dose 1*					
Subjects (N)	200			199	
Seropositivity rate % (95 % CI)	98.5	(95.7; 99.7)		96.0	(92.2; 98.2)
Seroconversion rate % (95 % CI)	95.0	(91.0; 97.6)		82.4	(76.4, 87.4)
GMC IU/mL (95 % CI)	6.8	(6.1; 7.5)		4.2	(3.8; 4.7)
*1 month after Dose 2*					
Subjects (N)	200			198	
Seropositivity rate % (95 % CI)	100	(98.2; 100)		100	(98.2; 100)
Seroconversion rate % (95 % CI)	100	(98.2; 100)		100	(98.2; 100)
GMC IU/mL (95 % CI)	424	(375; 480)		336	(296; 381)

ELISA: enzyme-linked immunosorbent assay, HPV: human papillomavirus, IgG: immunoglobulin G, CI: confidence interval, GMC: geometric mean concentration, N: number of subjects.

**Fig. 3:**
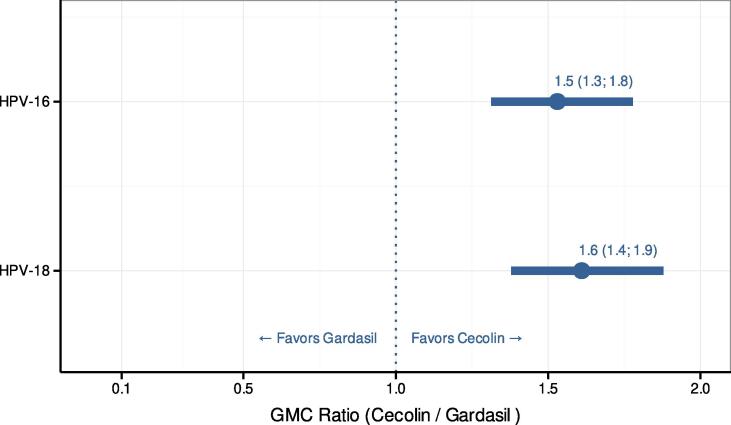
**Geometric mean concentration ratios at 6 months after Dose 1** HPV-16 and HPV-18 geometric mean concentration (GMC) ratios 6 months after Dose 1 between Cecolin 0, 6-month schedule and Gardasil 0, 6-month schedule with 95 % confidence intervals.

Six months after one dose of Cecolin for HPV-16, all participants (100 %) were seropositive and the seroconversion rate was 98.5 %; for HPV-18, all but 3 subjects (98.5 %) were seropositive, and the seroconversion rate was 95 %. Six months after one dose of Gardasil for HPV-16, all but 1 subject (99.5 %) were seropositive, and the seroconversion rate was 96.9 %. For HPV-18, all but 8 subjects (96 %) were seropositive, and the seroconversion rate was 82.4 %. IgG GMC (ELISA) 6 months after one dose of Cecolin and Gardasil were 18 and 12, respectively, for HPV-16, and 7 and 4, respectively, for HPV-18 (Table 2). The GMC ratio (Cecolin over Gardasil) was 1.5 (95 % CI [1.3; 1.8]) for HPV-16, and 1.6 (95 % CI [1.4; 1.9]) for HPV-18 (Fig. 3).

**Fig. 4:**
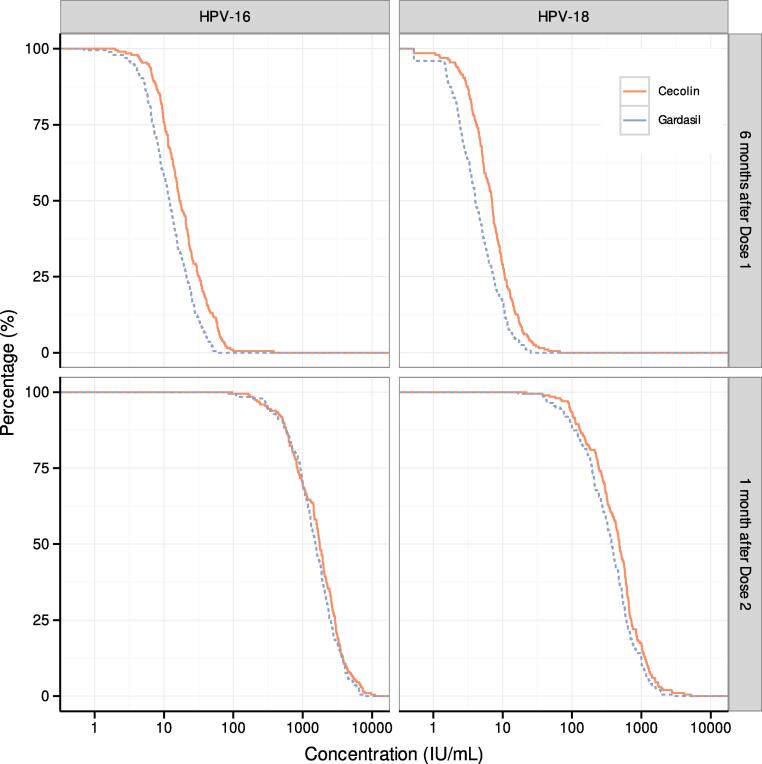
**Reverse cumulative distribution of immunoglobulin G HPV-16 and HPV-18 antibody concentrations by type-specific ELISA** Distribution of HPV-16 and HPV-18 enzyme-linked immunosorbent assay (ELISA) antibody concentrations 6 months post–Dose 1 and one month post–Dose 2 in the per protocol population. The percentage represents the proportion of the population with antibody levels equal to or above the concentration in x-axis.

The reverse distribution curves for IgG antibody levels by ELISA for each antigen and time point are shown in Fig. 4. Participants’ HPV-16 and HPV-18 IgG antibody concentrations 6 months post–Dose 1 and 1 month post–Dose 2 (type-specific ELISA) are shown in Supplemental Fig. 1.

Results for neutralizing antibodies using the PBNA by antigens for both groups are in line with the results by ELISA and are shown in Table 3, and the reverse distribution curves are shown in Supplemental Fig. 2.

**Table 3:** PBNA HPV-16 and HPV-18 IgG antibody response (per protocol population, PBNA subset).

	Cecolin 0, 6-month			Gardasil 0, 6-month	
**PBNA HPV-16**					
*6 months after Dose 1*					
Subjects (N)	40			40	
Seropositivity rate % (95 % CI)	97.5	(86.8; 99.9)		90.0	(76.3; 97.2)
Seroconversion rate % (95 % CI)	92.5	(79.6; 98.4)		77.5	(61.5; 89.2)
GMT titer (95 % CI)	110	(84; 143)		77	(58; 102)
*1 month after Dose 2*					
Subjects (N)	40			40	
Seropositivity rate % (95 % CI)	100	(98.2; 100)		100	(91.2; 100)
Seroconversion rate % (95 % CI)	100	(91.2; 100)		100	(91.2; 100)
GMT titer (95 % CI)	17,639	(12486; 24920)		16,056	(11878; 21704)
**PBNA HPV-18**					
*6 months after Dose 1*					
Subjects (N)	40			41	
Seropositivity rate % (95 % CI)	92.5	(79.6; 98.4)		97.6	(87.1; 99.9)
Seroconversion rate % (95 % CI)	75.0	(58.8; 87.3)		75.6	(59.7, 87.6)
GMT titer (95 % CI)	77	(49; 120)		50	(41; 61)
*1 month after Dose 2*					
Subjects (N)	40			41	
Seropositivity rate % (95 % CI)	97.5	(86.8; 99.9)		100	(91.4; 100)
Seroconversion rate % (95 % CI)	97.5	(86.8; 99.9)		100	(91.4; 100)
GMT titer (95 % CI)	6424	(3828; 10778)		7425	(5545; 9943)

PBNA: pseudovirion-based neutralization assay, HPV: human papillomavirus, IgG: immunoglobulin G, N: number of subjects, CI: confidence interval, GMT: geometric mean titer.

The frequency of reported AEs, overall, solicited local or systemic reactions, and unsolicited AEs occurred at similar rates between the two groups (Table 4). Pain at the injection site was the most frequently reported local solicited event, occurring overall in 2.0 % and 5.9 % of participants within the 30 min following Cecolin and Gardasil administration, respectively, and in 63.9 % and 67.3 % of participants within the 7 days following Cecolin and Gardasil administration, respectively. Headache was the most frequently reported systemic solicited event, occurring overall in 3.9 % and 2.4 % of participants within the 30 min following Cecolin and Gardasil administration, respectively, and in 18.5 % and 23.9 % of participants within the 7 days following Cecolin and Gardasil administration, respectively. Most solicited AEs were of mild intensity. One participant reported pain of grade 3 intensity one day following the first dose of Cecolin and two instances of grade ≥ 3 (≥39.3 °C) fever following Gardasil administration occurred, both in the context of an intercurrent infection (one instance was concurrent to an upper respiratory tract infection and the other to an episode of malaria). Solicited events were short-lived, with a median duration of two days for local and one day for systemic solicited reactions. An increase in local reactogenicity was observed following the second dose for both vaccines.

**Table 4:** Safety outcomes (total vaccinated population).

	Cecolin 0, 6-month schedule			Gardasil 0, 6-month schedule	
	(N = 205)			(N = 205)	
	n	Percentage (95 % CI)		n	Percentage (95 % CI)
**Any injection site or systemic reactions (within 7 days of vaccination of any dose)**					
Any reaction	146	71 (65; 77)		155	76 (69; 81)
Severe or greater	1	0.5 (0; 2.7)		2	1 (0.1; 3.5)
**Injection site (within 7 days of vaccination of any dose)**					
Any reaction	131	64 (57; 70)		138	67 (60; 74)
Severe or greater	1	0.5 (0; 2.7)		0	0 (0; 1.8)
**Systemic reaction (within 7 days of vaccination of any dose)**					
Any reaction	72	35 (29; 42)		76	37 (30; 44)
Severe or greater	0	0 (0; 1.8)		2	1 (0.1; 3.5)
**Unsolicited AE**					
Any non-serious AE (within 30 days of vaccination of any dose)	43	21 (16; 27)		52	25 (20; 32)
Any serious AE (within 30 days of vaccination of any dose)	1	0.5 (0; 2.7)		0	0 (0; 1.8)
Any serious AE (within 7 months after first dose)	1	0.5 (0; 2.7)		1	0.5 (0; 2.7)
Any related serious AE (within 7 months after first dose)	0	0 (0; 1.8)		0	0 (0; 1.8)
Any AE leading to withdrawal (up to data lock for interim analysis)	0	0 (0; 1.8)		0	0 (0; 1.8)

N: number of participants, n: number of participants with at least one event, CI: confidence interval, AE: adverse event.

Unsolicited AEs were reported in 21.0 % and 25.4 % of Cecolin and Gardasil recipients, respectively. All were considered not related to study vaccines and the majority of events fell under the infections and infestations system organ class of the MedDRA classification. Two serious AEs were reported up to the data freeze for the interim safety analysis—one case of pyrexia with no focus identified 26 days following the second dose of Cecolin, and one instance of snake bite 63 days following the second dose of Gardasil. Both events resolved and were assessed as not related to study vaccines.

## 4 Discussion

Cecolin prelicensure clinical trials demonstrating its efficacy and immunobridging to a pre-adolescent population were conducted in China [11], [12]. The present study is the first study to generate safety and immunogenicity data in a broader geographic representation, particularly in low-resource populations, where affordable vaccination programs are needed. Cecolin is indicated as a three-dose (0, 1, 6-month) regimen in women 15–45 years of age and as a two-dose (0, 6-month) or a three-dose (0, 1, 6-month) regimen in girls 9–14 years of age. Our study compares the immunogenicity of the indicated (0, 6-month) schedule of Cecolin as well as of two-dose extended (0, 12-month and 0, 24-month) schedules of Cecolin to Gardasil (0, 6-month regimen). Further, one of the groups will provide data on vaccine interchangeability. Flexibility of schedules (either spacing of doses or mixed-vaccine schedules) could address logistical issues encountered in vaccination programs by increasing programmatic flexibility. This study also assesses persistence of the immune response following one dose of Cecolin with the prospect that a single-dose regimen will simplify the vaccination schedule and potentially increase HPV vaccine uptake.

We report here on an interim immunogenicity and safety analysis conducted on the two vaccine groups receiving a 0, 6-month schedule and its related co-primary objective of assessing immunological non-inferiority of Cecolin versus Gardasil when administered on a 2-dose regimen 6 months apart. Data on extended and mixed dosing schedules, as well as immunogenicity 12 and 24 months following one dose of Cecolin, will be assessed at study end and reported in a future publication.

Cecolin obtained licensure in China in 2019 and WHO prequalification in 2021. The licensure was based on demonstration of high vaccine efficacy against high-grade genital lesions and persistent infections related to HPV-16 and HPV-18 in women aged 18–45 years [11]. The efficacy in a pre-adolescent population could subsequently be inferred since the immunogenicity of the vaccine in girls aged 9–17 years and 9–14 years receiving three or two doses, respectively, was shown to be non-inferior to that of young adult women aged 18–26 years [12]. Our study is the first evaluation of Cecolin compared to a widely used HPV vaccine and the first evaluation of Cecolin in LMICs of two geographical regions—South Asia and sub-Saharan Africa. Immunological non-inferiority of Cecolin on a 0, 6-month regimen to Gardasil was demonstrated for HPV-16 and HPV-18, the two oncogenic HPV types responsible for the vast majority of cervical cancer and HPV-related cancers at other anatomical sites. At one month after the second dose, all study participants were seropositive and had seroconverted (i.e., showed a four-fold increase in antibody concentrations from baseline by ELISA for HPV-16 and HPV-18).

In populations for which demonstration of clinical efficacy is not technically feasible, demonstration of immunological non-inferiority is widely accepted and referred to as immunobridging [13]. The non-inferiority criterion used in this study of a lower bound of the CI for the GMC ratio to be greater than 0.5 has been used in several trials assessing HPV vaccines and found acceptable to regulators [12], [14], [15], [16]. A non-inferiority criterion with a lower bound of 0.67 has also been used for HPV vaccine development, making it worth noting that the lower bound of the CIs for the GMC ratio of Cecolin to Gardasil was 0.89 for HPV-16 and 1.02 for HPV-18 and, therefore, would have met this more conservative criterion.

A good correlation between ELISA and PBNA has been established. Therefore, the IgG-binding antibody response is used as a high-throughput assay in the assessment of the immune response for HPV vaccines [10], [17]. In a recent study, the performance of seven HPV-16/18 serological assays (including type-specific ELISA and PBNA) was evaluated in 530 serum samples from participants having received single- or multi-dose regimens of Cervarix or Gardasil up to 36 months post-vaccination [9]. Both assays were deemed adequate to monitor immune response in the context of modest antibody levels, as observed following HPV single-dose vaccination at peak as well as at a time when the antibody plateau phase is reached several years after vaccine administration. The high correlation (Pearson 0.87 for HPV-16 and 0.95 for HPV-18) between ELISA and PBNA indicates that the ELISA can be used as a proxy for neutralization. Nevertheless, in our study we complemented the assessment by ELISA with the testing by PBNA in a subset (20 %) of participants (i.e., approximately 40 subjects per group). Type-specific neutralizing antibody results were in line with results using ELISA. One month after the second dose of vaccine, all study participants were seropositive by PBNA for HPV-16, and all but one Cecolin recipient were seropositive for HPV-18.

As anticipated and previously described, antibody levels following one dose were lower than following a multi-dose schedule [10], [16], [18]. Six months following one dose of either vaccine, seropositivity rates by ELISA were high. This finding was consistent with findings from previous trials [12], [16]. In addition, our study also assessed seroconversion rates following one dose (i.e., the proportion of subjects showing a four-fold increase in antibody levels from baseline). Seroconversion rates by ELISA were high for both antigens and ≥ 75 % of participants also seroconverted by PBNA. Findings six months following one dose of Cecolin administration compared favorably to those of Gardasil. Based on the antibody kinetic observed in other studies, the immune response is shown to remain stable from seven to 24 months following a single dose with either Gardasil 9 or Cervarix, or Gardasil [16], [18]. Therefore, we anticipate Cecolin to exhibit similar kinetics with stability of the antibody levels following one dose for several years. The ongoing study will generate additional data 24 months after one dose.

Evidence suggests that neutralizing antibodies represent the mechanism of protection; however, a minimum antibody level indicative of protection has not been established for HPV vaccines [19]. Despite lower antibody levels following a single dose, clinical protection against HPV-16 and HPV-18 infections provided by one dose is very high and similar to that conferred by two or three doses [10], [20]. Single-dose efficacy of Gardasil has been shown in the International Agency for Research on Cancer India trial with a vaccine efficacy of over 95 % up to 10 years post-vaccination [20]. Furthermore, in a recently published randomized, controlled trial in Kenya, vaccine efficacy against persistent HPV-16/18 infections was 97.5 % following a single-dose regimen of Gardasil 9 in women aged 15–20 years [21].

In the Dose Reduction Immunobridging and Safety Study, a randomized trial of different HPV vaccine schedules in Tanzanian girls 9–14 years of age, antibody responses after one dose of HPV vaccines were compared to those from observational HPV vaccine trials with demonstrated vaccine efficacy of one dose [22]. Relevant to our study, immunogenicity in girls 9–14 years of age measured 24 months after vaccination was shown to be non-inferior to the immunogenicity of one dose of Gardasil having been administered to girls 10–18 years of age and for which HPV-16 and HPV-18 efficacy up to ten years post-vaccination had been shown [3], [20]. Based on the body of evidence regarding protection conferred by Gardasil on a single-dose regimen, demonstrating a robust immune response following one dose of Cecolin in a magnitude that compares favorably to that of Gardasil is reassuring. Antibody levels, however, are known to reach a plateau phase 12–24 months after vaccination [10], [16], [18]. Although evaluations after a single dose of other HPV vaccines have shown comparable antibody levels at 6 months to later time points, continuing to monitor the immune response kinetics following Cecolin single-dose vaccination is important. In alignment with WHO’s recommendations for immunobridging, an evaluation of antibody persistence following one dose will occur 12 and 24 months after vaccination in our study and will be reported in a subsequent manuscript [8].

Cecolin was shown to have a safety profile very similar to that of Gardasil with a balanced frequency of safety outcomes in both study arms at the interim analysis. Up to study month 7, one serious AE each following Cecolin and Gardasil administration was reported. Both events were judged to not be related to study vaccine. Both vaccines were found to be well tolerated.

Strengths of our study include the inclusion of a diverse population of girls from a South Asian country and a sub-Saharan African country as well as the use of a product-agnostic antigen in the HPV serology assays, produced internally at the testing laboratory in an independent mammalian system by methods that are not specifically linked to either of the vaccine products. Immunogenicity assessments were performed using a validated HPV-16 and HPV-18 ELISA with high reproducibility that is maintained for the lower antibody ranges observed following HPV single-dose vaccination relative to multi-dose vaccination [9]. In addition, a qualified PBNA was utilized to assess functional neutralization responses, which was previously used as a reference assay for evaluating HPV single-dose immune responses [9]. Samples from both study groups were tested within the same batch to minimize variability and increase reliability of comparison between vaccines.

The study had an open-label design, which could be seen as a potential limitation with this type of study being prone to observer bias. The primary objectives, however, are based on immunological assessments performed in a blinded manner at the laboratory. One other limitation is that antibody levels might not have reached a plateau following one-dose administration at the timepoint for the interim analysis. Assessment of antibody persistence is ongoing, with data to be reported for later time points (12 and 24 months) following a single dose of Cecolin.

## 5 Conclusion

In summary, Cecolin in a 0, 6-month schedule was shown to be immunologically non-inferior to Gardasil in a population of girls 9–14 years of age from Bangladesh and Ghana. Cecolin elicits a robust antibody response after one and two doses. Compared to Gardasil, immunogenicity following one dose of Cecolin was similar up to six months post-vaccination.

The safety profile of Cecolin was similar to that of Gardasil and both vaccines were well tolerated. Cecolin obtained WHO prequalification in 2021 and, therefore, presents an affordable alternative for HPV vaccination. The safety and immunogenicity data from this pre-specified interim analysis in our study further support Cecolin use in LMICs.

## CRediT authorship contribution statement

**Khalequ Zaman:** Investigation, Writing – review & editing. **Anne E Schuind:** Conceptualization, Methodology, Writing – original draft, Writing – review & editing, Visualization. **Samuel Adjei:** Investigation, Writing – review & editing. **Kalpana Antony:** Writing – original draft, Writing – review & editing, Project administration. **John J Aponte:** Writing – original draft, Writing – review & editing. **Patrick BY Buabeng:** Investigation, Writing – review & editing. **Firdausi Qadri:** Investigation, Writing – review & editing. **Troy J Kemp:** Writing – original draft, Writing – review & editing, Investigation. **Lokman Hossain:** Investigation, Writing – review & editing. **Ligia A Pinto:** Investigation, Writing – review & editing. **Kristen Sukraw:** Writing – review & editing. **Niranjan Bhat:** Conceptualization, Funding acquisition, Writing – review & editing. **Tsiri Agbenyega:** Investigation, Writing – review & editing.

## Declaration of competing interest

The authors declare the following financial interests/personal relationships which may be considered as potential competing interests: Financial support for this study was provided by the Bill & Melinda Gates Foundation in Seattle, Washington, United States, and the Federal Ministry of Education and Research in Frankfurt, Germany. The findings and conclusions contained within are those of the authors and do not necessarily reflect positions or policies of the Bill & Melinda Gates Foundation. Financial support to icddr,b is provided by the governments of Bangladesh and Canada. This project has been funded in whole or in part with federal funds from the National Cancer Institute, National Institutes of Health, under Contract No. 75N91019D00024. The content of this publication does not necessarily reflect the views or policies of the Department of Health and Human Services, nor does mention of trade names, commercial products, or organizations imply endorsement by the United States Government. Aside from the funding provided for this study as described above, the authors do not have any other conflict of interest to declare.

## Data Availability

Data are from an interim analysis
