# Supplementary material for: Safety and immunogenicity of Innovax bivalent human papillomavirus vaccine in girls 9–14 years of age: Interim analysis from a phase 3 clinical trial
Source: Vaccine. 2024 Apr 2;42(9):2290–8. doi: 10.1016/j.vaccine.2024.02.077 (PMC11007388; doi:10.1016/j.vaccine.2024.02.077)
Supplement: Supplementary data 1 [file mmc1.docx]

**Appendix A**

Safety and immunogenicity of Innovax bivalent human papillomavirus vaccine in girls 9–14 years of age: interim analysis from a Phase 3 clinical trial

Authors

Khalequ Zaman, Anne Schuind, Samuel Adjei, Kalpana Antony, John J Aponte, Patrick BY Buabeng et al.

**Assay description:**

HPV-16 and HPV-18 virus-like particles (VLPs) and pseudovirion (PsV) particles were produced in a mammalian cell system, independent of the vaccine manufacturer’s production system, as previously described in detail (Tsang et al., 2020) with a few minor changes. Briefly, to produce HPV-16 and HPV-18 VLPs, used for ELISA testing, 293TT cells were transfected with codon-modified HPV-16 L1 plasmid DNA (p16L1h, provided by Dr. John Schiller) or were transfected with codon-modified HPV-18 L1L2 plasmid DNA (p18sheLL, provided by Dr. John Schiller) using Transporter 5® (Polysciences, Warrington, PA). To produce PsV particles, used in PsV-based neutralization assay (PBNA) testing, 293TT cells were transfected with either p16sheLL or p18sheLL (plasmids provided by Dr. John Schiller) encoding HPV type-specific L1 and L2 proteins and plasmid pYSEAP (plasmid provided by Dr. John Schiller) encoding secreted alkaline phosphatase (SEAP) using Lipofectamine™ 2000 (Thermo Fisher Scientific, Waltham, MA) (Tsang et al., 2020).

HPV Binding Antibody Measurement

The HPV-specific ELISA (binding antibody assay) microtiter plates (Thermo Scientific™ Nunc™ MaxiSorp, Thermo Fisher Scientific, USA) are coated with HPV-16 or HPV-18 VLPs and incubated at 4°C for three to five days. The plates are then washed and incubated with a blocking buffer containing 4% skim milk and 0.2% Tween 20 in phosphate-buffered saline. Next, the plates are washed and incubated with serially diluted participant serum. The plates are washed again, and a solution of peroxidase-labeled goat anti-human immunoglobin G (IgG) is added. After a final plate wash, the plates are developed with a tetramethylbenzidine substrate solution, and the reaction is stopped with 0.36 N sulfuric acid. Finally, the absorbance is measured with a microtiter plate reader (450 nm [λ1] and 620 nm [λ2], λ1 minus λ2). Antibody levels, expressed as ELISA units (EU)/mL, are calculated by interpolation of optical density values from the standard curve. The lower limit of quantitation of the HPV-16 assay was < 8 EU/mL, and the lower limit of quantitation of the HPV-18 assay was < 7 EU/mL. The results were converted to international units (IU)/mL based on the following conversion factor for HPV-16 (1 IU/mL = 5.66 EU/mL) and HPV-18 (1 IU/mL = 6.65 EU/mL).

HPV Neutralizing Antibody Measurement

The PBNA was set up by seeding 293TT cells in 96 well plates. Serially diluted participant serum and controls were incubated with HPV-16 or HPV-18 PsV containing the SEAP reporter plasmid and then added to the plated 293TT cells for 72 hours. The cell culture supernatant was harvested and evaluated with the Great EscAPe™ SEAP assay kit (Clontech Laboratories, Mountain View, CA). Neutralization titers were calculated by linear interpolation and defined as the reciprocal of the dilution that caused 50% reduction in SEAP activity compared with control. The reported neutralization titers reflect the mean value of duplicate testing for each sample. The lower limit of quantitation of the HPV-16 PBNA was a titer of < 21, and the lower limit of quantitation of the HPV-18 PBNA was a titer of < 16.

**Reference:**

Tsang SH, Basu P, Bender N, Herrero R, Kemp TJ, Kreimer AR, et al. Evaluation of serological assays to monitor antibody responses to single-dose HPV vaccines. Vaccine. 2020;38(38):5997–6006. doi:10.1016/j.vaccine.2020.07.017.

**Immunogenicity results**

**Supplementary Figure 1
HPV-16 and HPV-18 IgG participants’ antibody concentrations six months post–Dose 1 and one month post–Dose 2 (type-specific ELISA)**


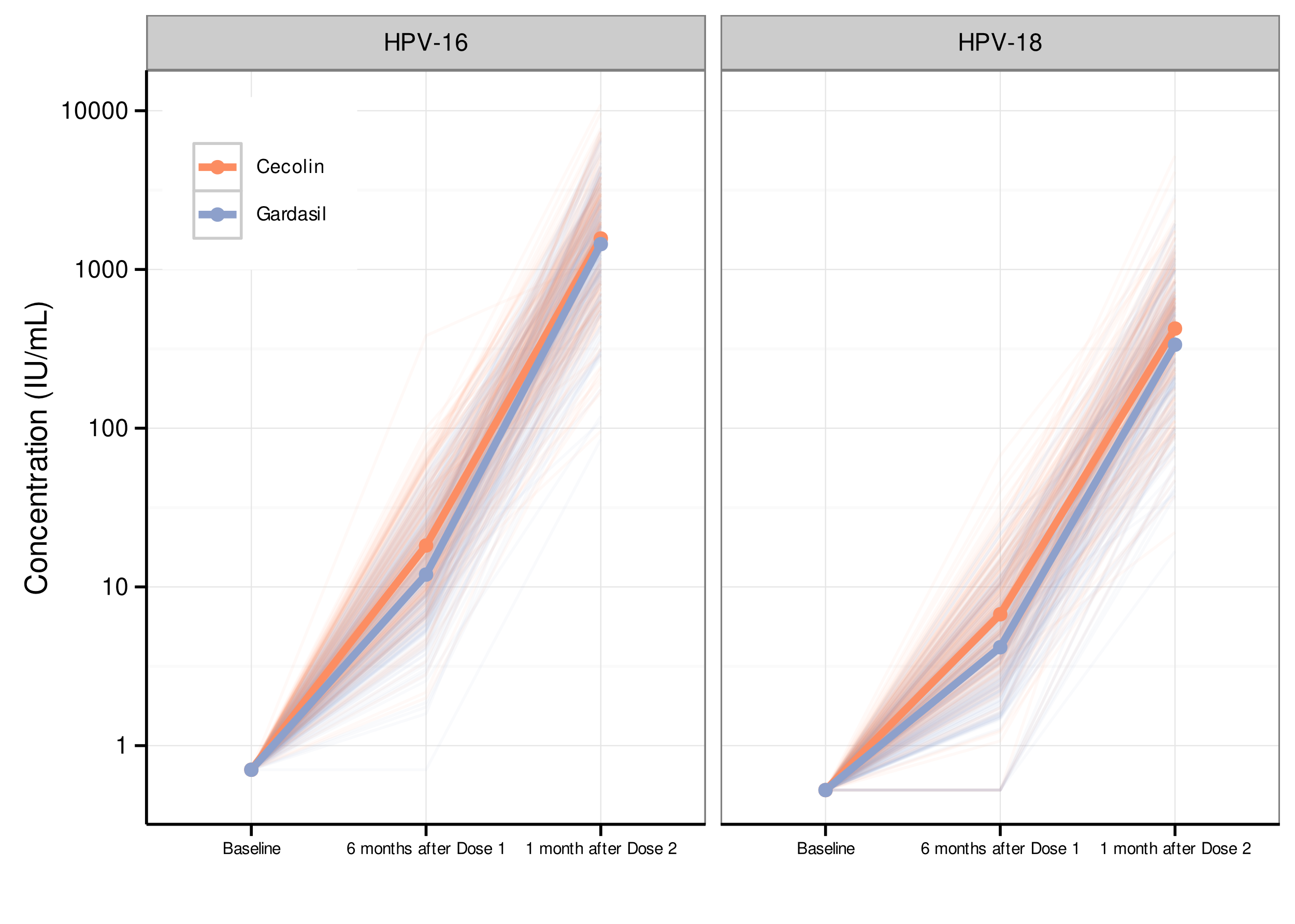


Legend

HPV-16 and HPV-18 IgG antibody concentrations six months post–Dose 1 and one month post–Dose 2 (type-specific ELISA). Note that the time points are not scaled to represent real time between time points. The thin lines represent individual data from each participant. The thick lines represent the geometric mean concentrations.

**Supplementary Figure 2**
**Reverse cumulative distribution of neutralizing HPV-16 and HPV-18 antibody titers by PBNA**

**
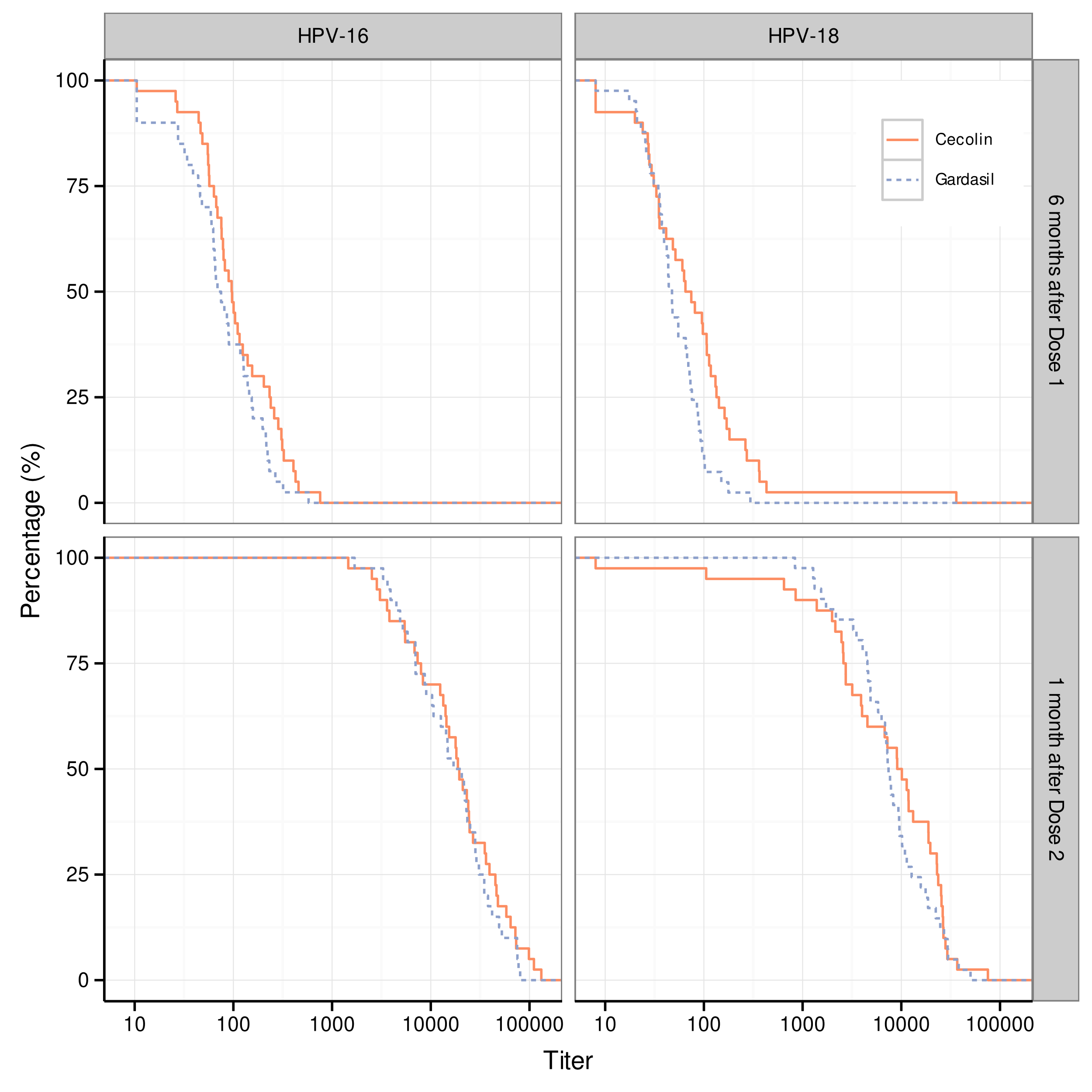
**

Legend:

Distribution of HPV-16 and HPV-18 PBNA antibody titers six months post–Dose 1 and one month post–Dose 2 in the per protocol population. The percentage represents the proportion of the population with antibody levels equal to or above the concentration in x-axis.
